# Identification of the Main Intermediate Precursor of l-Ergothioneine Biosynthesis in Human Biological Specimens

**DOI:** 10.3390/molecules21101298

**Published:** 2016-09-28

**Authors:** Salvatore Sotgia, Arduino A. Mangoni, Mauro Forteschi, Rhys B. Murphy, David Elliot, Elisabetta Sotgiu, Gianfranco Pintus, Ciriaco Carru, Angelo Zinellu

**Affiliations:** 1 Department of Biomedical Sciences, School of Medicine, University of Sassari, Sassari 07100, Italy; mforteschi@gmail.com (M.F.); eli.sotgiu@alice.it (E.S.); gpintus@uniss.it (G.P.); carru@uniss.it (C.C.); azinellu@uniss.it (A.Z.); 2 Department of Clinical Pharmacology, School of Medicine, Flinders University and Flinders Medical Centre, Adelaide SA 5042, Australia; arduino.mangoni@flinders.edu.au (A.A.M.); rhys.murphy@flinders.edu.au (R.B.M.); david.elliot@flinders.edu.au (D.E.); 3 Department of Biomedical Sciences, College of Health Sciences, Qatar University, Doha 2713, Qatar; 4 Quality Control Unit, University Hospital Sassari (AOU), Sassari 07100, Italy

**Keywords:** aminothione, capillary electrophoresis, mass spectrometry, quaternary ammonium, dismutation

## Abstract

A capillary electrophoresis coupled to tandem mass spectrometry (CE–MS/MS) has been used to make a qualitative determination of hercynine—the main precursor of l-ergothioneine biosynthesis—in some key human biological specimens, such as urine, whole blood, plasma, and saliva. From semiquantitative analysis results, the highest concentrations of hercynine were detected in saliva and whole blood, whereas much lower concentrations were measured in urine and plasma. Whole blood was the biological matrix with the highest concentration of l-ergothioneine followed by plasma, saliva, and urine. The antioxidant effects attributed to l-ergothioneine, along with its peculiar antioxidant mechanism, offer a possible explanation for the presence of the hercynine, as well as its concentration, in the considered biological matrices.

## 1. Introduction

**Figure 1:**
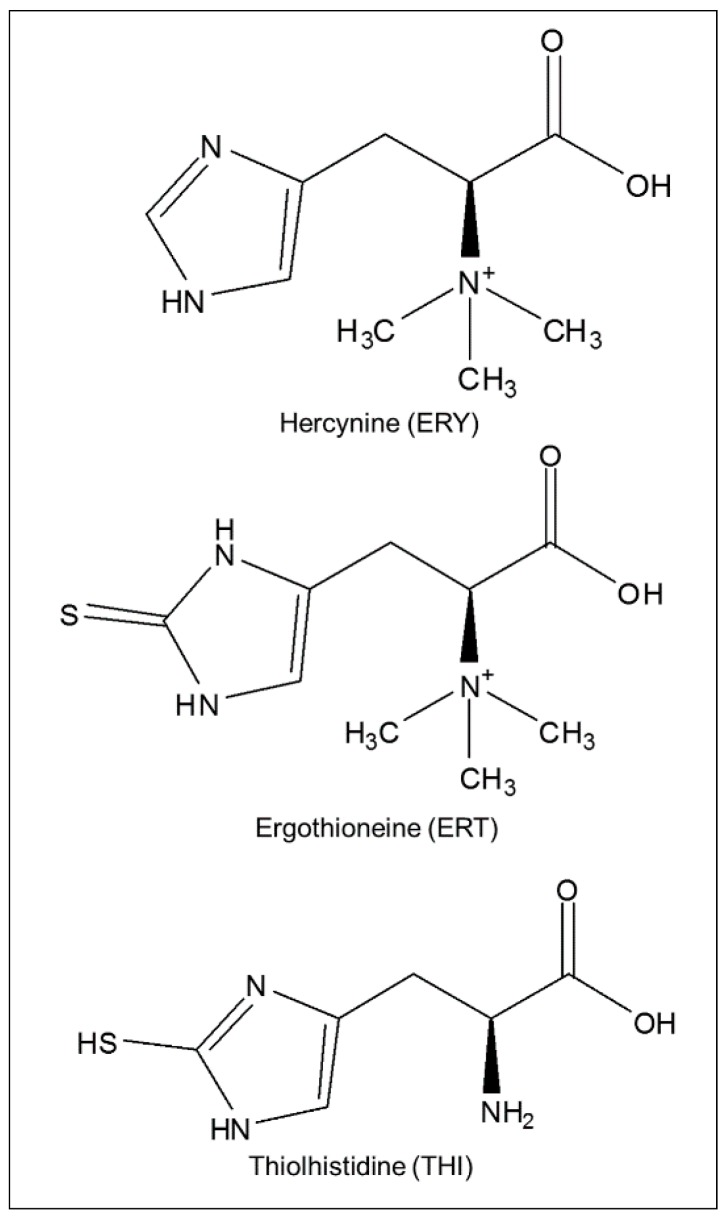
Chemical structures of analytes and internal standard.

l-ergothioneine (ERT; 2-mercaptohistidine trimethylbetaine) is an unusual hydrophilic low-molecular-weight thiol biosynthesized exclusively by some fungi, including edible mushrooms, and bacteria such as mycobacteria and cyanobacteria [1,2,3]. In mammals, ERT is acquired solely by dietary sources [1,4]. Through a specific organic protein transporter (ETT), ERT accumulates in mitochondria, cells, and tissues normally exposed to oxidative stress and involved in the inflammatory response process [1,3]. Unexpected high levels of ERT in red blood cells has been observed in some autoimmune disorders [5,6], such as rheumatoid arthritis and Crohn’s disease, in pre-eclamptic women [7] and, recently, in amniotic fluid of pregnant sheep after transfer of vitrified/thawed in vitro produced embryos [8]. Similar to glutathione (GSH), several observations suggest that ERT might act as antioxidant and scavenger of oxidizing species [9]. The antioxidant effect of ERT is generally described as part of a cycle where, following oxidation to disulphide or to mixed disulphide, ERT is regenerated by disulphide reduction, as is the case for other alkylthiols [10,11]. However, due to the tautomeric equilibrium of ERT between thiol and thione forms, with the latter predominant at physiological pH [12], ERT shows a peculiar stability and reactivity compared to other naturally occurring alkylthiols [13]. Consequently, the mechanism through which ERT performs its antioxidant effect differs from that of alkylthiols. As outlined in the past by Melville [14] and recently demonstrated by Servillo et al. [15], ERT can indeed oxidize to disulphide (ESSE). However, because of ESSE instability at physiological pH—and contrary to other disulphides—ESSE undergoes a progressive decomposition by disproportionation, rather than a reductive reconversion to ERT [15,16]. As a result, without reducing agents, ESSE can both generate hercynine (ERY) and regenerate ERT [15]. ERY, a betaine derived from histidine (see Figure 1), is the biological precursor of ERT [17]. Although ERY has been found in association with ERT in some organisms and biological matrix such as mushrooms, king crab, erythrocytes of cattle, and seminal fluid of boar [14], its distribution in living organisms is virtually unknown [18].

Similar to ERT, mammals cannot synthetize ERY. Therefore, the presence of ERY in the body might be ascribed to ERT oxidation. Notably, ETT shows lower affinity for ERY vs. ERT [19]. Therefore, ERY is more easily excreted by cells whereas ERT is avidly accumulated [15,20]. Thus, the determination of ERY in biological fluids could provide significant advantages over ERT in detecting oxidative stress states involving ERT. To our knowledge, however, no studies have specifically determined the occurrence of this imidazole derivative in human biological specimens. For this purpose, considering the lack of published analytical methods for its determination, the development of an ad hoc assay is needed. In this context, ERY might potentially show the same shortcomings of other betaine-like compounds. The quaternary moiety confers, in fact, a polar character to ERY. This might prevent a good chromatographic separation on reversed-phase columns, without the use of ion-pairing reagents as additives. In addition, the optical features of ERY, principally due to the imidazole ring, might not be enough to measure relatively low concentrations. It may therefore be necessary to develop a derivatization reaction to improve the limit of detection and the limit of quantification. Alternatively, field-amplified sample injection (FASI) capillary electrophoresis could overcome both the chromatographic and the sensitivity issues. However, it must also be considered that ERY is not commercially available, hence the need for in-house synthesis. In this context, the specificity and sensitivity of mass spectrometry technology provides undeniable advantages over other analytical options. Using this approach, synthesized ERY can be detected without an extensive purification step. In the same manner, ERY can also be identified at high sensitivity in the samples. Thus, following this strategy, we used a capillary electrophoresis coupled to tandem mass spectrometry (CE–MS/MS) to make a qualitative determination of ERY in key human biological specimens, such as urine, whole blood, plasma, and saliva.

## 2. Results and Discussion

Both ERY and ERT were detectable in all the human samples examined (Table 1).

Semiquantitative analysis revealed that the highest concentrations of ERY were detected in saliva and whole blood, whereas much lower concentrations were measured in urine and plasma. As expected, the highest concentration of ERT was detected in whole blood, with lower concentrations in plasma, saliva, and urine. The relatively high concentrations of ERT in whole blood vs. other body fluids, primarily due to accumulation in red blood cells (RBCs) [21], further supports its almost exclusive intracellular distribution and poor tissue turnover [12]. ERT stems from erythroblasts that, like other myeloid precursor cells, strongly express the ETT transporter [13]. Thus, ERT is high in the early life of RBCs and, because of the inability of mature RBCs to take up additional ERT, declines as the age of erythrocytes increases. Under normal conditions, however, ERT drops only very slowly over the entire erythrocyte life span [13,14]. Although the physiological role of ERT is not yet fully elucidated, it might serve as a powerful catalytic scavenger of oxidizing species that are not free radicals [13,22]. In particular, as observed in in vitro experiments, ERT in erythrocytes reduces ferryl hemoglobin thus avoiding its detrimental effect on promoting autocatalytic oxidation of native hemoglobin to methemoglobin [13,23]. The observation of the synchronized uptake of ERT and iron by means, respectively, of the ETT and the transferrin receptor CD71 [13]—both heavily expressed in nucleated erythrocyte progenitor cells—provides further support for this role. Regardless of the biological role of ERT, the unstable ESSE appears to be the main product of its oxidation. Dismutation of ESSE, in turn, leads both to ERY production and to reduction back to ERT [15]. In particular, from 2 mol of ESSE, 3 mol of ERT and 1 mol of ERY are formed [15]. The concentrations of the latter in whole blood, therefore, could be a marker of the redox activity of ERT. Thus, according to the semiquantitative analysis of ERY (Table 1) and to the stoichiometry of redox reaction [15], the indirect evaluation of the ESSE shows whole blood concentrations of about 2.6 µmol/L. In turn, ERT concentrations from the hydrolytic breakdown of ESSE were approximately 4 µmol/L, while the ERT involved in the redox process was about 8% (~5.2 µmol/L) of its total amount in the RBCs. Although data in literature are not available for a comparison, these small concentrations are consistent with physiological conditions, as expected in the apparently healthy subjects enrolled in this study. Baseline conditions can also explain the relatively low concentrations of ERY detected in plasma, which likely represent the balance between cellular efflux and excretion in urine. With a whole blood-to-plasma ratio of 9, our data suggest that the efflux of ERY from RBCs occurs slowly. It is difficult to establish whether this unexpected finding can be generalized for other types of cells. If it was, it could be explained by the interaction of ERY with ETT. Although the ETT-mediated transport of ERY is 25-fold less than ERT [19], it might still be enough to reduce cellular efflux. However, as ETT is not functional in mature RBCs [13,14], this hypothesis is less plausible for this cell type. It has been speculated that ERY is an unstable metabolite with a short half-life, decomposing spontaneously into histidine and histamine [24]. Therefore, ERY plasma concentrations may be affected by this metabolic route. The urine-to-plasma ratio of 3, instead, would support the hypothesis of renal clearance. Unlike ERY, the urine-to-plasma ratio of ERT was 0.15. This is not surprising considering that ETT is abundantly expressed in the kidneys [13], where presumably it allows the reabsorption of ERT. More difficult to interpret are the data on the ERT and ERY content in saliva. Unlike other compartments, such as RBCs or the kidney, salivary glands do not express ETT [12,13]. Therefore, ERY and ERT are likely to be generated in neighboring organs and tissues expressing ETT, such as the trachea, lungs and, to a lesser extent, the esophagus [12,13,19]. High expression of ETT, however, also occurs in skin fibroblast and keratinocytes [25]. Therefore, it is also possible that similar cells of the oral mucosa express ETT. In this scenario, ERT and ERY in saliva could derive from the typical exfoliative processes involving the epithelium of the oral mucosa, or from the presence of traces of blood. Other authors have previously ruled out the potential role of the microbiota, at least for ERT, in animals [26].

## 3. Material and Methods

### 3.1. Chemicals

ERT and ammonia (25%) were purchased from Vinci-Biochem (Florence, Italy) and Merck (Darmstadt, Germany), respectively. Methanol (HPLC grade), l-2-thiol-histidine (THI), ammonium acetate, iron(III) chloride hexahydrate (FeCl_3_·6H_2_O), and formic acid were obtained from Sigma Aldrich Italy (Milan, Italy). All buffer solutions were filtered through a disposable 0.22 µm membrane filter purchased from Merck Millipore Italy (Milan, Italy). Disposable ultrafiltration devices, Vivaspin 500 Micro Concentrators, were obtained from Sigma Aldrich Italy. High-purity water, obtained from a Millipore Milli-Q system, was used throughout the experiments (Merck Millipore Italy).

### 3.2. Collection and Processing of Biological Samples

Twelve human biological samples from three healthy volunteers with no medical history—three for each type (whole blood, plasma, urine, saliva)—were processed for the qualitative analysis by CE–MS/MS. Prior to the analysis, all samples were spiked with a 65 µmol/L THI solution, used as structural analogues internal standard (IS), then filtered on Vivaspin 500 Micro Concentrators to remove proteins and molecules up to 10 kDa. For this purpose, 500 µL of each sample were added to 10 µL of internal standards and mixed thoroughly by vigorous vortex-mixing. The samples were then filtered by centrifugation of the filter devices at 21,380× *g* for 30 min at room temperature. By contrast, prior to filtration, the whole blood samples were lysed by hypotonic shock with cold water in a sample/water ratio of 1:5. Then, after vortex-mixing, 500 µL of supernatant was processed as above. All subjects gave their informed consent for inclusion before they participated in the study. The study was conducted in accordance with the Declaration of Helsinki, and the protocol was approved by the Ethics Committee of Azienda Asl n°1 di Sassari (2262/CE).

### 3.3. Hercynine Synthesis

**Figure 2:**
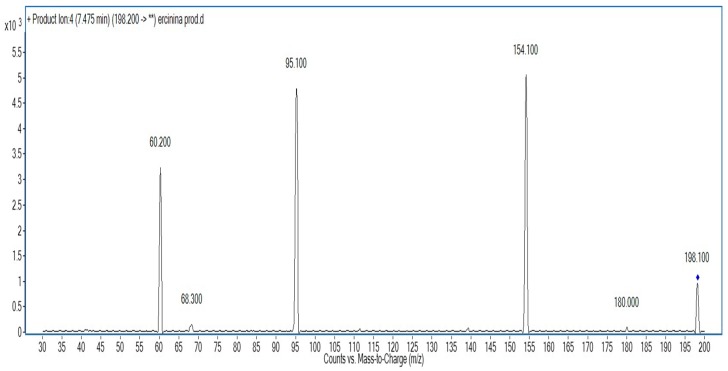
MS/MS pattern of synthesized hercynine (ERY).

Because of the commercial unavailability of ERY, to confirm its identification in the biological samples, ERY was prepared from ERT by oxidation with ferric chloride as described by Barger and Ewin [27], with some adjustment. Briefly, 5 mg of ERT was mixed with 20 mg of FeCl_3_·6H_2_O dissolved in 1 mL of water. The mixture was initially heated for 30 min in a thermoblock heater, and then it was cooled to room temperature. Without further purification, the structure was confirmed by CE–MS/MS, and a peak at *m*/*z* 198.1 was considered consistent with the parent ion peak [M + H]^+^ of ERY [18]. The structure was further ascertained by MS/MS experiments showing that, in agreement with previous reports, the main fragment ions were at *m*/*z* 60.2, 95.1, and 154.1 [18] (see Figure 2).

### 3.4. Semiquantitative Analysis

Mass detection was accomplished in positive ion mode by multiple reaction monitoring (MRM) of the precursor–product ion transitions, *m*/*z* 198.1→95.2, 230→127.2, 188.1→142.2 for ERY, ERT, and THI, respectively. Determination of appropriate MRM transitions and the optimization of parameters, such as fragmentor voltage or collision energy, was conducted by MassHunter Optimizer Software (Agilent Technologies Italy, Milan, Italy). For this purpose, a background electrolyte (BGE) containing a different analyte each time was used to get a constant concentration of compound in the electrospray. Since ERY was used as synthesized without further purification, a semiquantification was performed by comparing the area of a known amount of IS spiked to the samples vs area of the ERY or ERT in the samples. All of the analyses were performed in triplicate.

### 3.5. CE Equipment and Electrophoretic Conditions

CE−MS/MS experiments were performed by using an Agilent 7100 Capillary Electrophoresis System (CE) fitted with a built-in diode-array detector, an autosampler, an air pressure pump, and a 30 kV power supply with a current range up to 300 µA. The CE system was coupled via the Agilent coaxial sheath-liquid sprayer interface with an Agilent MSD mass spectrometer 6460 Series equipped with a triple-stage quadrupole analyzer operated with the Agilent G1948B electrospray ionization (ESI) source. An Agilent 1200 series isocratic LC pump was used for the sheath-liquid delivery to the sprayer. CE system operation, MS/MS control, data acquisition, and analysis were run by the Agilent MassHunter Workstation Software (all Agilent Technologies Italy). Electrophoresis was performed on an uncoated fused-silica capillary (Beckman Coulter Italy, Milan, Italy). The total/effective capillary length (to the MS inlet) was 100 cm (inner diameter 75 µm) and injection volume was 11.26 nL (50 mbar × 3 s) for an injection plug length of 4.38 mm. The separation was carried out at 20 °C and 30 kV (10 µA) at normal polarity with an overpressure of 0.1 psi (35 mbar) from inlet to outlet. The BGE was a 5 mmol/L ammonium acetate buffer adjusted with formic acid at pH 6. After each run, the capillary was equilibrated with a 1:1 mixture of ammonia/H_2_O for 1 min, H_2_O for 1 min, then with BGE for 1 min. To provide a stable electrical connection between the tip of the capillary and the ground, a sheath-liquid consisting of a 1:1 mixture of 5 mmol/L ammonium acetate/methanol was delivered isocratically at a flow rate of 6 µL/min to the sprayer through the 1:100 flow splitter of the LC pump. The capillary voltage in the mass spectrometer was set at 4 kV, dry nitrogen gas was heated to 300 °C and delivered at a flow rate of 10 L/min, while the nitrogen nebulizing gas (N_2_) pressure was 10 psi.

## 4. Conclusions

This is one of the few reports on the identification of ERY in biological samples. Since no ERY standard was commercially available, to overcome an expensive time-consuming purification step, a capillary electrophoresis coupled to tandem mass spectrometry was used to detect ERY by the known exact *m*/*z* value of 198.1 and its fragmentation pattern at *m*/*z* 60.2, 95.1, and 154.1. While aware of its analytical accuracy limitations, this strategy was used to perform a semiquantitative analysis of ERY and ERT by using THI as a structural analogue internal standard. Both ERT and ERY were detectable in all the studied samples, at concentrations consistent with physiological conditions. Although at this stage of the study an alimentary intake of ERY cannot be excluded with certainty, the antioxidant effects attributed to ERT offer a possible explanation for the presence of the ERY and ERT, as well as their concentrations, in the specimens considered in this study. The relatively high concentrations of analytes in the saliva suggest that this easily collectable biological specimen might be used to further investigate the biological and pathophysiological roles of ERY and ERT, as well as the potential use of ERY as a marker of the redox activities involving ERT.

During the review process of this work, Cheah et al. [28] reported nanomolar levels of ERY in whole blood, plasma, and urine. No investigation was performed on the concentrations in saliva. The semiquantitative approach used in this work can account for concentrations higher than those reported by the study of Cheah et al. which, on the whole, strengthens our conclusions.

## Figures and Tables

**Table 1:** Concentrations of the analytes in the human biological samples. Values are the mean of three replicates (average of nine readings per type).

Samples	ERT (µmol/L ± SD)	ERY (µmol/L ± SD)
Whole Blood (*N* = 3)	66 ± 2.2	1.3 ± 0.1
Plasma (*N* = 3)	1.1 ± 0.3	0.2 ± 0.1
Urine (*N* = 3)	0.2 ± 0.2	0.5 ± 0.3
Saliva (*N* = 3)	0.6 ± 0.2	2.2 ± 0.5
